# *Arabidopsis* KASH domains are differentially required for distinct LINC complex roles in stomata, roots and pollen

**DOI:** 10.1242/jcs.264672

**Published:** 2026-04-30

**Authors:** Lily A. Schumacher, Norman R. Groves, Daniel E. Conway, Iris Meier

**Affiliations:** ^1^ Department of Molecular Genetics, The Ohio State University, Columbus, OH 43210, USA; ^2^ Department of Biomedical Engineering, The Ohio State University, Columbus, OH 43210, USA

**Keywords:** *Arabidopsis*, LINC complex, SUN–KASH interaction, Nuclear movement, AlphaFold

## Abstract

Nuclear movement and positioning can be mediated by linker of nucleoskeleton and cytoskeleton (LINC) complexes, which consist of Sad1-UNC-84 (SUN) proteins and Klarsicht-ANC-1-Syne homology (KASH) proteins. KASH proteins bind SUN proteins via a short KASH domain. Unlike animal KASH domains, plant KASH domains are shorter, lack ability to form disulfide bonds and have a different C-terminal sequence motif. Here, we examined the specificity of KASH domains using two *Arabidopsis* KASH proteins, WIP1 and SINE1. We show experimentally that the SINE1 KASH domain is required for SINE1 function in stomata and the WIP1 KASH domain for WIP1 function in root hairs, but that the SINE1 and WIP1 KASH domains are interchangeable for the WIP1 role in nuclear movement in pollen tubes. Through molecular modeling, we found that SINE1 has two distinct binding modes that are dependent on its interaction partner (SUN1 or SUN2), whereas WIP1 binds very similarly to both SUN1 and SUN2. We propose that this requirement for specific KASH domains reflects differences between the SUN1–SINE1 and SUN1–WIP1 interaction models and might indicate a different tolerance of the interactions to force.

## Teaser

**Summary:** Plant KASH proteins with exchanged KASH domains were tested for *in vivo* function. Both specificity and interchangeability of KASH domains were found, and modeling suggests a structural explanation.

## INTRODUCTION

The nucleus is a dynamic organelle that undergoes a variety of nuclear movements, anchorage and shape changes that are required for development and environmental response in opisthokonts. In humans, the nucleus undergoes a long-distance migration during multiple stages of neuron development, maintains equal spacing as it moves along microtubules in muscle fibers and undergoes a basal nuclear movement during ear hair development (Lee and Burke, 2018). All these phenomena have been shown to involve linker of nucleoskeleton and cytoskeleton (LINC) complexes. LINC complexes are protein bridges that span the inner nuclear membrane (INM) and outer nuclear membrane (ONM).

INM Sad1-UNC-84 (SUN) and ONM Klarsicht-ANC-1-Syne homology (KASH) proteins form the core of the LINC complex, which connects to nuclear lamins at the nuclear periphery and to the cytoskeleton in the cytoplasm (Starr and Fridolfsson, 2010). Within the perinuclear space between the two membranes of the nuclear envelope (NE), the SUN domain of SUN proteins anchors KASH proteins to the NE via the KASH domain (Starr and Fridolfsson, 2010). The cytoplasmic regions of KASH proteins are highly variable, depending on the cellular function of the protein. The characteristic 8–32-amino-acid (aa) C-terminal KASH domain terminates in a conserved ‘PPPX’ motif (Cain et al., 2018; Sosa et al., 2012; Starr and Fridolfsson, 2010). Through this SUN–KASH interaction, LINC complexes can transduce forces from the cytoskeleton to the nucleus and across the NE (Jahed et al., 2015; Lombardi et al., 2011). Structural studies have indicated that human SUN2 forms a trimeric complex, with each KASH domain interacting with a groove, termed the KASH-binding pocket, formed by two adjacent SUN domains (Cain et al., 2018; Jahed et al., 2018; Sosa et al., 2012).

In *Caenorhabditis elegans*, both the length and structure of the KASH domain are important factors in LINC complex function. A nuclear migration defect observed in the KASH protein mutant *unc83* cannot be complemented by a modified version of UNC-83 where the KASH domain is extended by one amino acid (Cain et al., 2018). In the case of the KASH protein ANC-1, conserved cysteine residues in the KASH domain are required for its function in nuclear anchorage (Cain et al., 2018). KASH domain specificity for nuclear anchorage and nuclear movement has been tested by examining chimeric versions of UNC-83 and ANC-1 with swapped KASH domains. The ANC-1 KASH domain is insufficient to replace the UNC-83 KASH domain in nuclear movement (Jahed et al., 2019). In contrast, the UNC-83 KASH domain is partially functional in replacing the ANC-1 KASH domain for the nuclear anchorage function of ANC-1. Shortening the ANC-1 KASH domain to the length of the UNC-83 KASH domain also affects ANC-1 function (Jahed et al., 2019). These findings indicate differences in the requirements for KASH domain length and amino acid composition depending on the specific function of the respective LINC complex. The authors proposed that the relatively brief interactions required for nuclear positioning in the case of UNC-83 might require less resilience to tensile force than the continuous connections to actin involved in nuclear anchoring (Cain et al., 2018; Jahed et al., 2019).

Plant LINC complexes and LINC complex-associated factors are substantially different from their animal counterparts (Meier et al., 2017). Plant SUN proteins (SUN1 and SUN2) were identified through homology with human SUN proteins but diverge in their nucleoplasmic region (Graumann, 2014; Graumann et al., 2010; Murphy et al., 2010; Starr and Fridolfsson, 2010). Unlike SUN proteins, plant KASH proteins appear to have no shared origin with animal KASH proteins, with unrelated cytoplasmic domains and shorter KASH domains (9–13 aa compared to 8–32 aa for opisthokonts) that terminate in a consensus ‘(V/I)PT’ motif, distinct from the terminal ‘PPPX’ consensus motif of animal KASH proteins (Poulet et al., 2017; Zhou and Meier, 2013; Zhou et al., 2012, 2014).

WPP-Interacting Protein (WIP)-type KASH proteins are conserved in basal angiosperms, monocots and dicots (Poulet et al., 2017). In *Arabidopsis thaliana*, *WIP1*, *WIP2* and *WIP3* are a family of three related, redundantly acting genes. WIPs form a complex with ONM WPP-interacting tail-anchored proteins 1 and 2 (WIT1 and WIT2) (Zhao et al., 2008). The WIT–WIP–SUN complex is required for elongated nuclear shape in root hairs and trichomes, nuclear positioning in trichomes,and nuclear movement in mature root hairs, in conjunction with myosin XI-i (Tamura et al., 2013; Zhou et al., 2012, 2015a). In pollen, the WIT–WIP–SUN complex is important for nuclear movement in elongating pollen tubes and male fertility (Moser et al., 2020; Zhou and Meier, 2014; Zhou et al., 2015b).

SUN-interacting nuclear envelope proteins (SINEs), which are present in dicots, monocots, gymnosperms and club mosses, are the evolutionarily oldest KASH proteins in plants (Poulet et al., 2017). SINE1 and SINE2, two paralogous *Arabidopsis* KASH proteins, were both identified as important factors in abscisic acid (ABA)-induced stomatal closure (Biel et al., 2020a). During ABA-induced stomatal closure, two paired guard cells undergo several cellular changes, including reorganization of both the F-actin and the microtubule cytoskeletons, vacuolar fragmentation and fusion, ion influx and water uptake (Biel et al., 2020b, 2022). In both the *sine1-1* and *sine2-1* insertional mutants, guard cells are hyposensitive to the hormone ABA (Biel et al., 2020a). SINE1, which has been shown to colocalize with actin, is required for repolymerization of the actin cytoskeleton during stomatal closure (Biel et al., 2022; Zhou et al., 2014). SINE2 is involved in F-actin depolymerization during stomatal closure (Biel et al., 2022). Aside from *Arabidopsis*, homologs of SINE1 and SINE2 have also been functionally characterized in *Medicago truncatula* and in *Zea mays* (named MLKS proteins) (Gumber et al., 2019a,b; Newman-Griffis et al., 2019; Poulet et al., 2017; Zhou et al., 2014).

Here, we have tested whether different plant KASH domains are specific to cellular and physiological LINC complex functions. To this end, we expressed chimeric SINE1 and WIP1 KASH proteins with swapped KASH domains in the respective mutant plant backgrounds. The data show that the SINE1 KASH domain is required for SINE1 function in stomata and the WIP1 KASH domain for WIP1 function in trichoblasts, the precursor to root hairs. In contrast, the SINE1 and WIP1 KASH domains are interchangeable for the WIP1 role in nuclear movement in pollen tubes. Whereas pollen nuclear movement depends on both SUN1 and SUN2, root hair nuclear shape only depends on SUN1 (Zhou et al., 2015a,b). Modeling *Arabidopsis* LINC complexes using AlphaFold suggests that the SINE1 KASH domain adopts a strong binding mode with SUN1 that is distinct from that for WIP1. We propose that this distinct binding mode of the SINE1 KASH domain to SUN1 cannot reproduce the root hair nuclear shape function of WIP1 in the WIP1^SINE1KASH^ mutant. Specificity of KASH domains, dependent on cellular roles and structural constraints, could indicate the importance of force transfer in plant LINC complex function.

## RESULTS

### Generation of KASH domain-swap chimeras

**Fig. 1.**
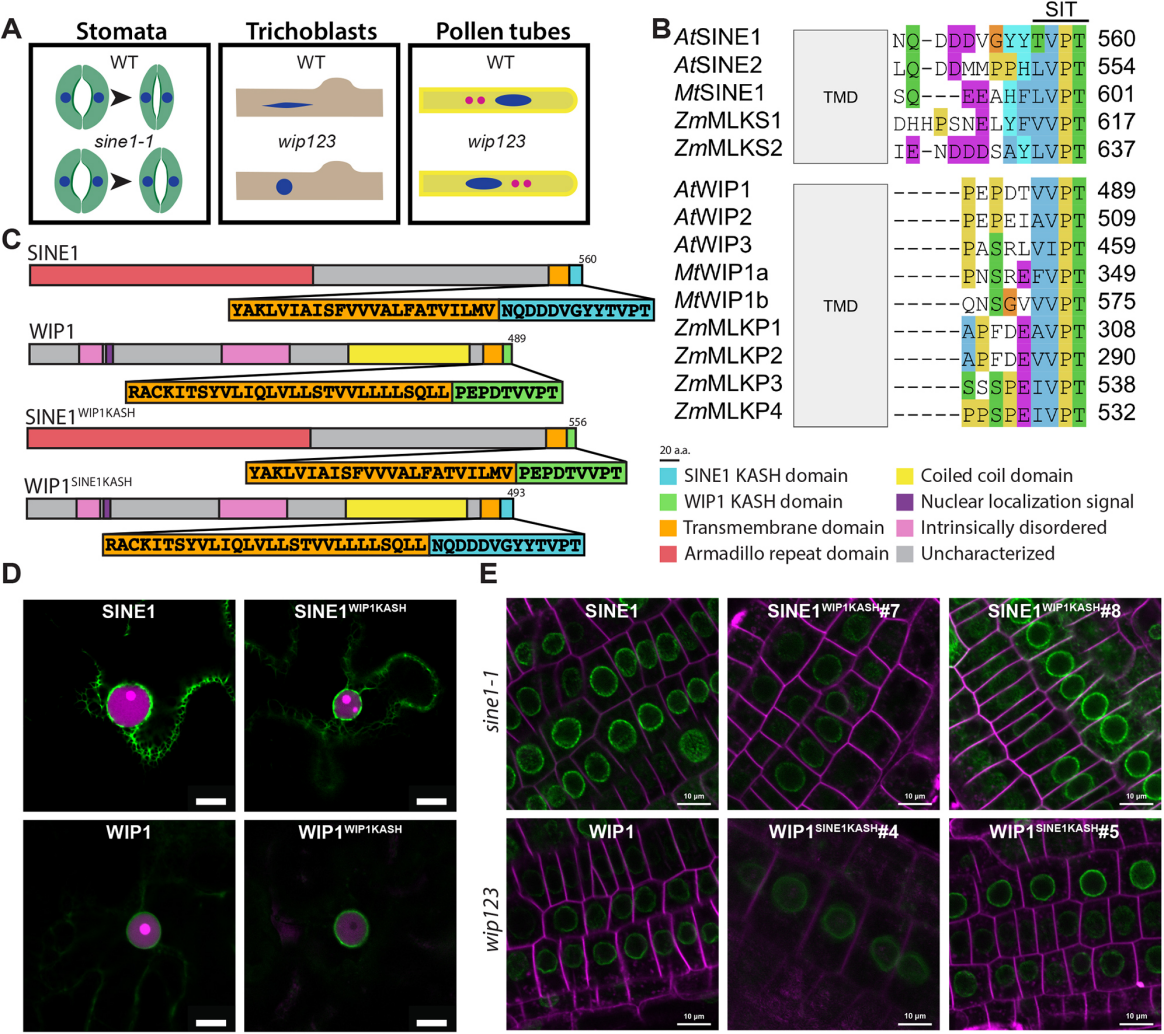
**KASH domain swap experimental design and NE localization.** (A) *Arabidopsis* KASH protein mutant phenotypes in stomata, trichoblasts and pollen tubes. Nuclei, dark blue; sperm cells, pink. (B) Multispecies alignment of plant KASH domains. Sequences were aligned using Clustal Omega. Sequences are colored according to Clustal X coloring threshold. SIT, SUN-interacting tail; TMD, transmembrane domain. (C) Gene diagram of SINE1, WIP1, SINE1^WIP1KASH^ and WIP1^SINE1KASH^. Known protein domains of SINE1 and WIP1 are depicted. Pop-outs show the TMD (orange) and KASH domains (cyan and green). Domains are drawn to scale and colored according to the key to the right. (D) Localization of GFP–SINE1, GFP–WIP1, GFP–SINE1^WIP1KASH^ and GFP–WIP1^SINE1KASH^ transiently expressed in *N. benthamiana* leaf epidermal cells*.* Magenta, nucleus labeled with H2B–mCherry. (E) Localization of GFP–SINE1, GFP–WIP1, GFP–SINE1^WIP1KASH^ and GFP–WIP1^SINE1KASH^ in transgenic *Arabidopsis* root tips. GFP–SINE1 and GFP–SINE1^WIP1KASH^ in *sine1-1*, GFP–WIP1 and GFP–WIP1^SINE1KASH^ in *wip123*. Magenta, plasma membrane counterstained with FM4-64. Images in D and E are representative of 10 and 15 experimental repeats, respectively. Scale bars: 10 μm.

WIP1, WIP2 and WIP3 are involved in nuclear shape determination in root and root hair cells, and in nuclear movement in root hairs and pollen tubes (Fig. 1A) (Tamura et al., 2013; Zhou and Meier, 2014; Zhou et al., 2012). SINE1 and SINE2 play a role in regulating stomatal closure in leaves (Fig. 1A) (Biel et al., 2020a; Zhou and Meier, 2014). We constructed a multisequence alignment of plant KASH domains across *Arabidopsis*, *M. truncatula,* and *Z. mays* (Fig. 1B). SINE-type KASH domains are longer at around 13 aa, whereas WIP-type KASH domains are shorter at 9 aa. All investigated KASH domains contained a C-terminal SUN-interacting tail (SIT) motif consisting of a branched nonpolar amino acid (V or I) followed by PT (Zhou et al., 2012, 2014). SINE KASH proteins had a conserved negative charge patch of aspartic acid and glutamic acid residues, followed by an aromatic residue prior to the terminal (V/I)PT. Most WIP KASH domains started with one or two rigid proline residues and ended with the terminal (V/I)PT motif. To assess the functional similarities and differences between plant KASH domains, we chose WIP1 and SINE1 as representative plant KASH proteins.

To explore these functional and structural differences between SINE and WIP KASH domains, two KASH domain-swap chimeras were designed (Fig. 1C). The SINE1^WIP1KASH^ chimera contains the cytoplasmic domain of SINE1, including its N-terminal Armadillo repeat-like domain, and the transmembrane domain, but the WIP1 KASH domain has been substituted for the SINE1 KASH domain. The WIP1^SINE1KASH^ chimera contains the WIP1 cytoplasmic domain, including the WIP1 intrinsically disordered regions, nuclear localization signal and coiled coil domain, as well as the transmembrane domain, and the SINE1 KASH domain in place of the WIP1 KASH domain (Fig. 1C).

N-terminal GFP fusion proteins of SINE1^WIP1KASH^ and WIP1^SINE1KASH^ were generated, with the constitutive CaMV 35S promoter driving expression. GFP–SINE1^WIP1KASH^ and GFP–WIP1^SINE1KASH^ were transiently expressed in *Nicotiana benthamiana* leaf epidermal cells to determine whether they are targeted to and retained at the NE, indicating that they are inserted into the membrane and bound by the inner NE SUN proteins (Zhou et al., 2012, 2014). Under these conditions, GFP–WIP1^SINE1KASH^ was almost exclusively associated with the NE, and was indistinguishable from GFP–WIP1 (Fig. 1D). GFP–SINE1 has previously been shown to associate with filamentous structures in the cytoplasm, likely F-actin, both under overexpression conditions in transient transformation, and in some cell types in transgenic *Arabidopsis* plants (Zhou et al., 2014). GFP–SINE1^WIP1KASH^ faithfully recapitulated this localization pattern, with the majority of GFP–SINE1 and GFP–SINE1^WIP1KASH^ decorating the NE (Fig. 1D). We conclude from this that both chimeric proteins are targeted to the NE, and in the case of SINE1, a secondary cytoplasmic location, consistent with their interaction with *N. benthamiana* SUN proteins, as shown previously for the wild-type (WT) proteins (Zhou et al., 2014).

Next, stably transformed *Arabidopsis* lines were generated – SINE1pro::GFP-SINE1^WIP1KASH^ in the *sine1-1* background and WIP1pro::GFP-WIP1^SINE1KASH^ in the *wip123* background (Xu et al., 2007; Zhou et al., 2014). All tested chimeric proteins localized to the NE in root tip cells, indicating the chimeric KASH domains were able to associate with SUN proteins (Fig. 1E).

### The SINE1 KASH domain is required for SINE1 function during stomatal closure

**Fig. 2.**
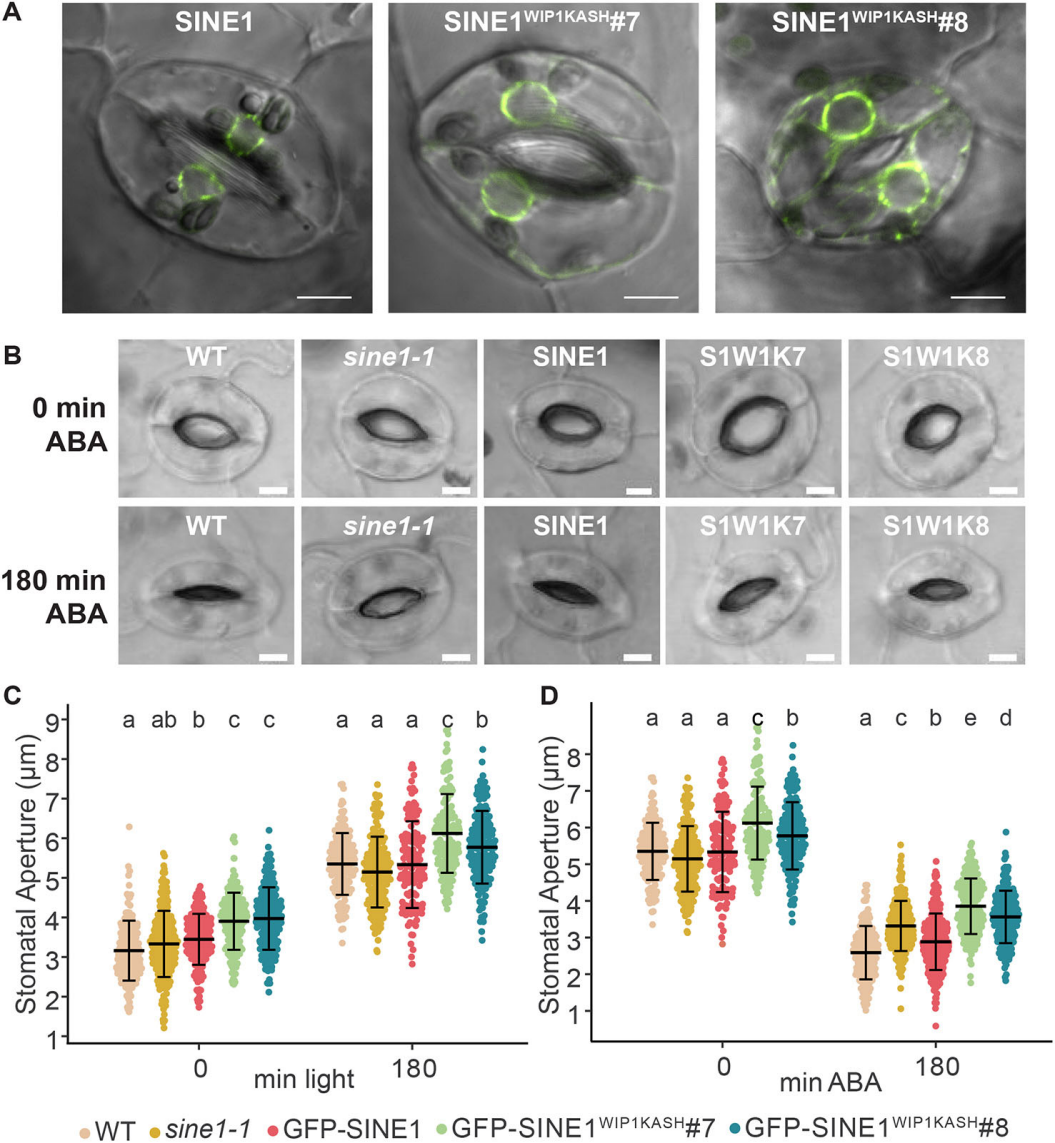
**The SINE1 KASH domain is required for stomatal dynamics.** (A) Localization of GFP–SINE1, GFP–SINE1^WIP1KASH^#7 and GFP–SINE1^WIP1KASH^#8 in stomata of transgenic *Arabidopsis* lines. Images representative of 15 experimental repeats. (B–D) Representative images (B) of 0 min and 180 min ABA-exposed stomata. S1W1 K, SINE1^WIP1KASH^. Leaves from 5–6-week-old short-day plants were placed in (C) opening buffer containing Ca^2+^ and K^+^ for 180 min then (D) transferred to closing buffer containing 20 μM ABA for 180 min in darkness. 180 min light and 0 min ABA are the same datapoints. Mean±s.d.; *N*>130 for each line, three biological repeats. Lowercase letters represent statistical groups different by *P*<0.05 from each other (two-way ANOVA paired with Tukey's HSD) (Table S3). Scale bars: 5 μm.

SINE1 is required for stomatal closure, with loss of SINE1 leading to an inability of stomata to fully close when induced by the drought-signaling hormone abscisic acid (ABA) (Biel et al., 2020a). GFP–SINE1 was located at the NE in the two nuclei of the paired guard cells that together form a stomate (Fig. 2A). GFP–SINE1^WIP1KASH^ similarly localized to the NE in guard cells in both transgenic lines (Fig. 2A).

The *sine1-1* mutant only has a marginal defect in opening stomata in the presence of opening buffer (see Materials and Methods) and white light (Biel et al., 2020a). In contrast, *sine1-1* is significantly impaired in closing the stomata in the presence of ABA (Biel et al., 2020a). To investigate the ability of GFP–SINE1^WIP1KASH^ to complement the *sine1-1* stomatal phenotype, both stomatal opening (Fig. 2C) and ABA-induced stomatal closing were tested (Fig. 2D). Immediately after removal from pre-incubation in the dark, the diameter of the stomatal pores at their widest distance was measured. As shown previously, WT, *sine1-1* and GFP–SINE1 in *sine1-1* all had relatively narrow diameters at 3–3.5 µm at 0 min light. After 3 h of white light treatment, all three lines reached an aperture of ∼5 µm. The two GFP–SINE1^WIP1KASH^ lines started out at a wider aperture of ∼4 µm, and after 3 h of white light they reached 5.5–6 µm (Fig. 2C). After 180 min of white light treatment, 20 µM ABA was added and leaves were moved to the dark. Thus, data for the 180 min light timepoint and the 0 min ABA timepoint in Fig. 2C and D are identical and only shown here separately for greater clarity.

After 180 min of ABA treatment, WT stomata closed to 2.5 µm whereas *sine1-1* stomata closed to only 3.2 µm on average, consistent with previously reported data (Biel et al., 2020a). GFP–SINE1 in *sine1-1* closed to a value similar to WT, demonstrating that the SINE1 mutant phenotype is complemented by the GFP fusion protein, as shown previously (Biel et al., 2020a). In contrast, the two SINE1^WIP1KASH^ lines closed to only 3.5–4 µm, indicating that the chimeric fusion protein cannot complement the *sine1-1* mutant, despite being located at the NE and containing an unaltered cytoplasmic domain. We noted that stomatal apertures in the GFP–SINE1^WIP1KASH^ lines were larger at all timepoints than stomata in all other genetic backgrounds tested and appeared larger than the other paired cells at 0 min of ABA (Fig. 2B–D; Fig. S1A,B). However, expressing this data as a percentage of the aperture at 180 min light, also showed reduced closing in the GFP–SINE1^WIP1KASH^ lines, confirming the result (Fig. S1C). To test whether the larger apertures were based on a developmental effect, such as larger cell size, the total area of stomata after 180 min of ABA treatment was measured. No significant difference in overall size was found (Fig. S1D). Although the reason for the overall wider aperture of SINE1^WIP1KASH^ is not known, the data indicate that the rate of closure of the two GFP–SINE1^WIP1KASH^ lines best matched *sine1-1*, therefore the SINE1 KASH domain is required for SINE1 function during stomatal closure.

### The WIP1 KASH domain is required for WIP1 function in trichoblast nuclear shape determination

**Fig. 3.**
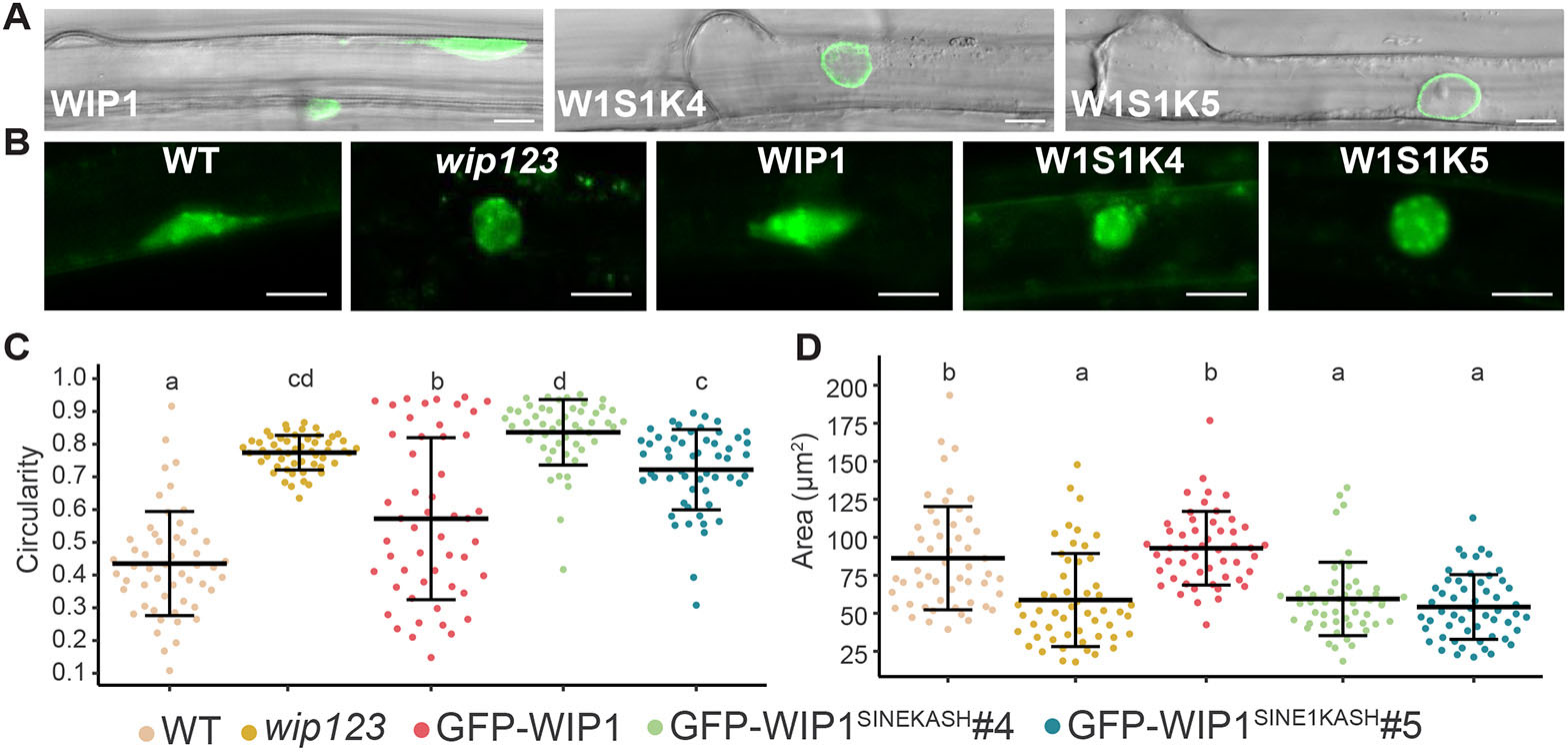
**The WIP1 KASH domain is required for trichoblast nuclear shape determination.** (A) Localization of GFP–WIP1, GFP–WIP1^SINE1KASH^#4 and GFP–WIP1^SINE1KASH^#5 in trichoblasts of transgenic *Arabidopsis* lines. Roots of 7-day-old seedlings. Images are representative of 50 experimental repeats. W1SK, WIP1^SINE1KASH^. (B) Representative SYBR green stained trichoblast nuclei for each line shown in C and D. (C,D) Graphs of trichoblast (C) circularity and (D) area. Mean±s.d.; *N*>50 for each line. Lowercase letters represent statistical groups different by *P*<0.05 from each other (one-way ANOVA paired with Tukey's HSD) (Table S4). Scale bars: 10 μm.

In *Arabidopsis*, several cell types show a polar nuclear migration that is relevant during development. One such cell type is developing root hairs, the single-cell extrusions of the root epidermis that function in water and mineral uptake in the soil. Root hairs develop from trichoblasts, a specific type of root epidermal cell. In trichoblasts, the nucleus adopts an inner polar position, away from the cell surface that faces the environment. Upon root hair development, this position shifts outward and the nucleus begins to migrate into the bulge initiating the developing root hair. The nucleus moves at a constant distance with the tip of the elongating root hair until the root hair is mature, upon which it becomes mobile and able to move back and forth throughout the root hair cell. The biological role of the latter movement is not known. Laser trap experiments have shown that nuclear positioning near the tip during root hair development is required for cellular elongation (Ketelaar et al., 2002; Nakamura et al., 2018). In trichoblasts, young root hairs and mature root hairs, the nucleus has a highly elongated shape (Figs 1A and 3A). It has been shown that in a *wip123* mutant (and a *wit12* mutant) root hair nuclei are round and are less mobile in fully mature root hairs (Tamura et al., 2013; Zhou et al., 2012, 2015a).

The directed movement of the nucleus in trichoblasts towards the developing root hair depends on actin (Nakamura et al., 2018). Although this observation has not been quantified, data (Nakamura et al., 2018) indicate that in several mutants with defects in trichoblast polar nuclear migration, the elongated shape of the nucleus is also lost. Given that the *wip123* mutant has more circular nuclei in mature root hairs, we investigated whether this change also occurs in trichoblasts, thus preceding root-hair elongation. Fig. 3B,C shows that trichoblast nuclei were significantly more circular in *wip123* than in WT, suggesting that the loss of nuclear elongation precedes the development of the root hair. We then examined whether expression of the two GFP–WIP1^SINE1KASH^ lines would complement the nuclear shape phenotype in trichoblasts. Fig. 3B,C shows that expressing GFP–WIP1 in *wip123* significantly reduced the circularity index to almost WT levels, whereas GFP–WIP1^SINE1KASH^#4 and GFP–WIP1^SINE1KASH^#5 expression in *wip123* had no effect on the circularity index. Fig. 3A shows that both GFP–WIP1^SINE1KASH^ lines express in range of the level of GFP–WIP1, with GFP–WIP1^SINE1KASH^#4 expression being slightly lower than that of GFP–WIP1^SINE1KASH^#5, and that the fusion proteins are correctly associated with the NE. To assess whether nuclear size was also affected in *wip123*, we measured the nuclear area in all five lines. Fig. 3D shows that indeed, *wip123* nuclei were on average smaller than WT nuclei, and that expression of GFP–WIP1 brought the nuclear size back to WT levels. In contrast, GFP–WIP1^SINE1KASH^#4 and GFP–WIP1^SINE1KASH^#5 expression in *wip123* had no effect on nuclear size. This shows that nuclei in both GFP–WIP1^SINE1KASH^ lines are more circular and smaller than WT and therefore the WIP1 KASH domain is required for the role of WIP1 in controlling the elongated shape of trichoblast nuclei.

### The WIP1 and SINE1 KASH domains are interchangeable for WIP1 function during male germ unit migration in the pollen tube

When *Arabidopsis* pollen grains germinate on the stigma of a flower, a single-cell pollen tube extends by tip-growth and protrudes through the style to reach the ovaries and release the sperm cells contained in the pollen grain. The nucleus of the pollen grain (vegetative nucleus, VN) journeys at a relative constant distance to the pollen tube tip and is connected by a cytoplasmic extension to the two sperm cells (SCs), which follow (the three elements together are called the male germ unit, MGU). In mutants of the WIT-WIP-SUN LINC complex (e.g. *wip123*), this process is interrupted, leading to an early reversal of the order of sperm cells and VN and a later distancing of the VN from the migrating sperm cells. Ultimately, unruptured pollen tubes and unfertilized ovules occur (Zhou and Meier, 2014; Zhou et al., 2015b). Here, we used the reversed transport order of the MGU as a signature phenotype of the *wip123* mutant to test whether chimeric GFP–WIP1^SINE1KASH^ is functional in this nuclear transport process.

**Fig. 4.**
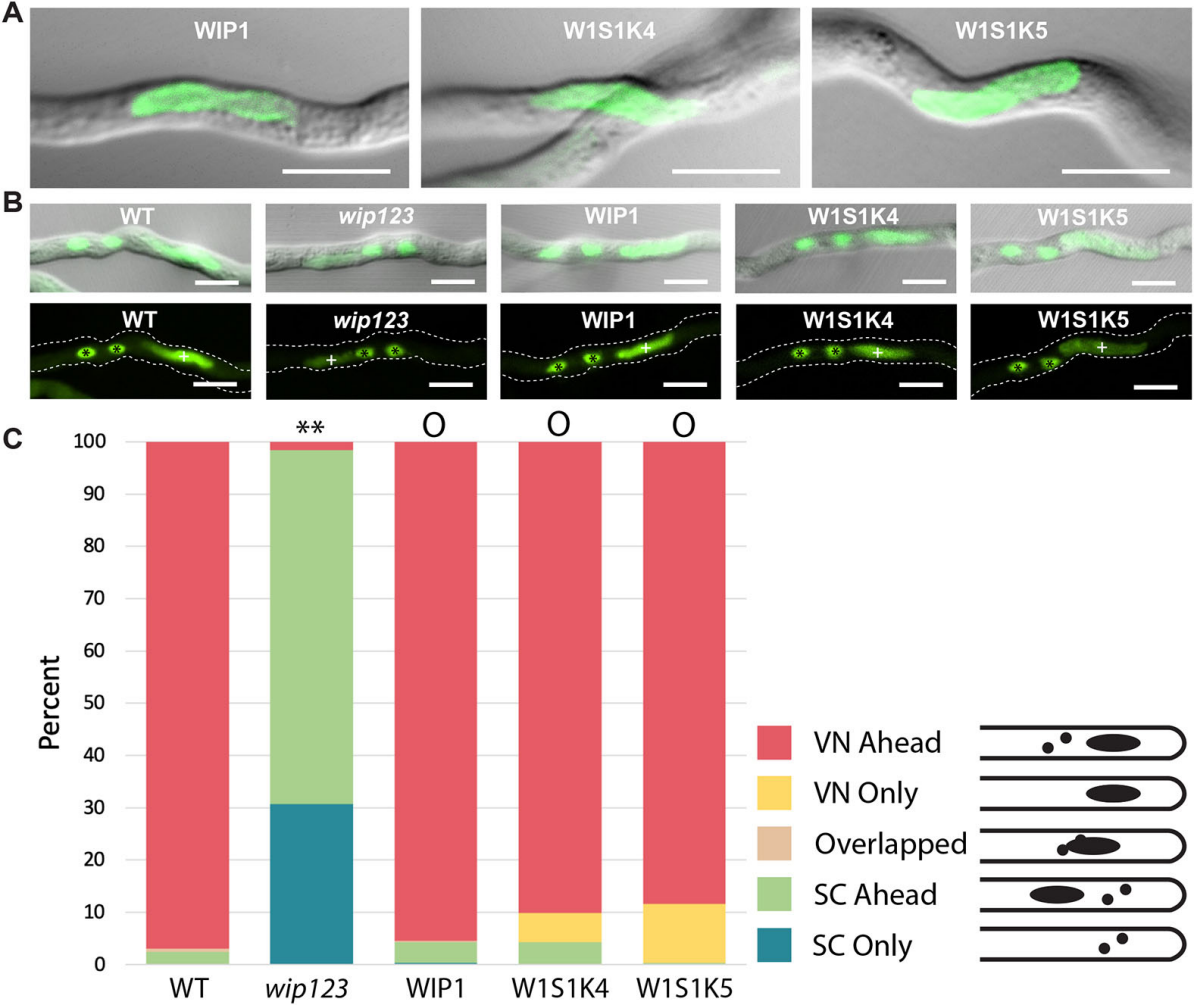
**The WIP1 and SINE1 KASH domains are interchangeable for male germ unit migration order.** (A) Localization of GFP–WIP1, GFP–WIP1^SINE1KASH^#4 and GFP–WIP1^SINE1KASH^#5 in pollen tubes of transgenic *Arabidopsis* lines. W1S1K, WIP1^SINE1KASH^. Images are representatives of 15 experimental repeats. (B) Representative images of SYBR green stained semi-*in vivo* pollen tubes for each line. Top DIC plus GFP; bottom GFP alone. Tips of pollen tubes point to the right. The white + indicates a vegetative nucleus; black * indicates sperm cell nuclei. The pollen tubes are outlined with dashed lines. (C) Graph of MGU order. Colored according to key to the right. *N*>150 for each line. Counts expressed as percentages of *N*. **P<0.01; ^O^*P*>0.05 (not significant) when compared with the WT (two-tailed Fisher's exact test). Scale bars: 10 μm.

 Fig. 4A shows that GFP–WIP1^SINE1KASH^ is expressed in the pollen tubes of both transgenic lines and that the fusion protein is located at the NE of the vegetative nucleus, as is GFP–WIP1, which localized similarly to what was found in previous characterization (Zhou and Meier, 2014). We used semi-*in-vivo* pollen tube germination and visualized the two sperm cell nuclei and the VN by SYBR green staining of the nucleic acids in pollen tubes and imaging 5 h post pollination (Fig. 4B). We defined five arrangements of the VN and SCs, as shown in Fig. 4C and quantified their relative abundance. In WT pollen tubes, the VN was ahead of the SCs in over 95% of the pollen tubes (pollen tube tips in Fig. 4B are oriented to the right). As shown previously (Zhou and Meier, 2014), the majority of MGUs in *wip123* showed the two SCs ahead and ∼30% of cases showed only the SCs, indicating that the VN was not in the field of imaging. GFP–WIP1 complemented this phenotype back to the WT situation. Unlike what was seen for root nuclear shape, the two transgenic lines expressing GFP–WIP1^SINE1KASH^ were almost as efficient in complementing the *wip123* mutation as GFP–WIP1 and statistically not different from WT (Fig. 4B,C). This suggests that WIP1^SINE1KASH^ can function in the correct transport of the MGU, while not being sufficient to provide the elongated nuclear shape of trichoblasts; in turn, this indicates that different mechanisms involving the KASH domain underly these two biological roles.

### Modeling *Arabidopsis* SUN–KASH interactions

Alphafold 3 (Abramson et al., 2024) was utilized to model the interactions of *Arabidopsis* KASH domains with SUN domains. In humans, SUN2 forms trimers, which can bind the KASH domain of three KASH proteins (Sosa et al., 2012; Wang et al., 2012), whereas the structure of plant LINC complexes is largely unknown. As an initial indicator of the accuracy of predicted *Arabidopsis* LINC complex structures, we used confidence scores (ipTM values) calculated by AlphaFold (<0.6 failed prediction, 0.6-0.8 moderate confidence,>0.8 high confidence) (Abramson et al., 2024; Jumper et al., 2021). *Arabidopsis* SUN1 and SUN2 as well as KASH proteins WIP1 and SINE1 are all transmembrane proteins. Models of the full proteins had low confidence scores, likely because of the absence of the lipid bilayer (Fig. S2A,B). Modeling the shortest region required for an interaction has been shown to be effective and give higher confidence models (Lee et al., 2024). Successfully modeling *Arabidopsis* SUN as a trimer, in analogy to the human SUN2–KASH structures, required the SUN domain and part of the coiled-coil domain (Fig. S2A,B). For KASH trimer structures, we used the KASH domain and transmembrane domain (TMD). We found that using too much of the SUN coiled-coil domains and structured KASH regions gave erroneous self-interactions, whereas just the SUN domain and KASH domain had too little structural context (Fig. S2A,B). To confirm the most likely multimer structure for *Arabidopsis* SUN–KASH interactions, we modeled one to six copies of each SUN–KASH pair and validated that three copies of each SUN and KASH protein gave the highest confidence (Fig. S2C) (Abramson et al., 2024; Jumper et al., 2021).

**Fig. 5.**
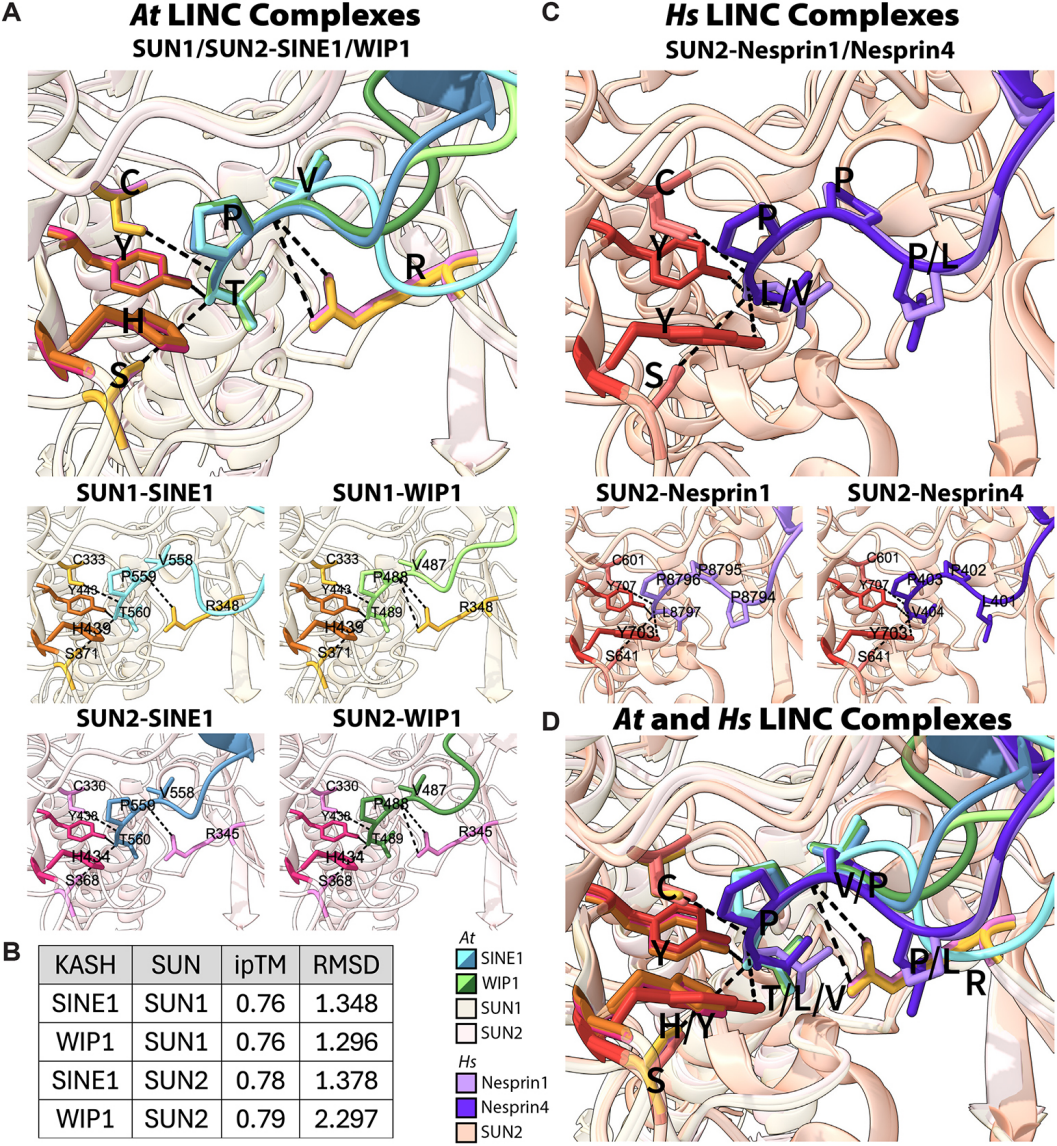
**KASH-binding pocket structural conservation.** (A) *Arabidopsis* (*At*) SUN–KASH binding pocket. SINE1 residues: V558, P559 and T560; WIP1 residues: V487, P488 and T489. Top, overlay of four different SUN–KASH pairs. Bottom, individual SUN–KASH binding pockets. Black dashed lines indicate H-bonding. Previously reported KASH-binding pocket residues for SUN1 are indicated in dark orange (H349 and Y443) and SUN2 in dark pink (H434 and Y438). Other KASH binding residues for SUN1 indicated in light orange (C333, S371 and R348) and for SUN2 in light pink (C330, S368 and R345). (B) Confidence measures for AlphaFold predictions. ipTM calculated by AlphaFold. RMSD compared KASH domain structure across five predictions of one SUN–KASH pair. (C) Human (*Hs*) SUN–KASH binding pocket. Nesprin-1 residues: P8794, P8795, P8796 and L8797; nesprin-4 residues: L401, P402, P403 and V404. Top, overlay of two different SUN–KASH pairs. Bottom, individual SUN–KASH binding pockets. Conserved SUN2 KASH-binding residues highlighted in peach (C601 and S641) or red (Y703 and Y707) in the same position as established *Arabidopsis* SUN-binding residues. Black dashed lines indicate H-bonding. (D) Overlay of four *At* SUN–KASH binding pockets and two *Hs* SUN–KASH binding pockets. Proteins colored according to the key at the bottom.

We next examined the terminal VPT residues of both SINE1 and WIP1, which are required for SUN–KASH binding, with WIP1ΔVVPT and SINE1ΔTVPT showing a diminished NE localization (Newman-Griffis et al., 2019; Zhou et al., 2012, 2014). SUN residues known to be required for KASH binding in humans have been shown to similarly be required in *Arabidopsis* SUNs (SUN1, H349 and Y443; SUN2, H434 and Y438) (Newman-Griffis et al., 2019; Zhou and Meier, 2013, 2014; Zhou et al., 2015b). Our modeling of *Arabidopsis* SINE1–SUN1, WIP1–SUN1, SINE1–SUN2 and WIP1–SUN2 interactions agreed with these studies, as these histidine and tyrosine residues are positioned deep in the KASH-binding pocket of SUN1 and SUN2 (Fig. 5A). H-bond analysis of the SUN–KASH interface revealed the SUN1 and SUN2 residues that bind the VPT, including the previously identified histidine and tyrosine, and other shared KASH-binding residues (SUN1, C333, S371 and R348; SUN2, C330, S368 and R345) (Table S2). We see that the KASH-binding pocket is highly conserved both in amino acid position and binding contacts in *Arabidopsis* (Fig. 5A). We note that WIP1 appeared to have an additional H-bond to R348 in SUN1 and R345 in SUN2 that SINE1 does not. Root mean square deviation (RMSD) values of ∼2 indicate low variation between the five predictions of each structure, indicating further confidence in the SUN–KASH models (Fig. 5B).

Next, we compared our *Arabidopsis* KASH-binding pocket models to the solved crystal structures of human SUN2 bound to the KASH domains of nesprin-1 or nesprin-4 (KASH1–SUN2, PDB code 4DXR; KASH4–SUN2, PDB code 6WMD; Fig. 5C) (Sosa et al., 2012; Wang et al., 2012). We found that human and *Arabidopsis* KASH-binding pockets showed strong structural overlap, despite the different lengths and terminal motifs (Fig. 5D). The only H-bonding differences we found were that *Arabidopsis* KASH proteins formed a bond with an arginine within this pocket, which human KASH does not, and human KASH formed a H-bond with both tyrosine residues in the pocket while the histidine residue in the same position in plant KASH does not. The confidence scores and visual validations of the known terminal binding motif confirmed to us that these structures are good predictions of *Arabidopsis* SUN–KASH interactions.

**Fig. 6.**
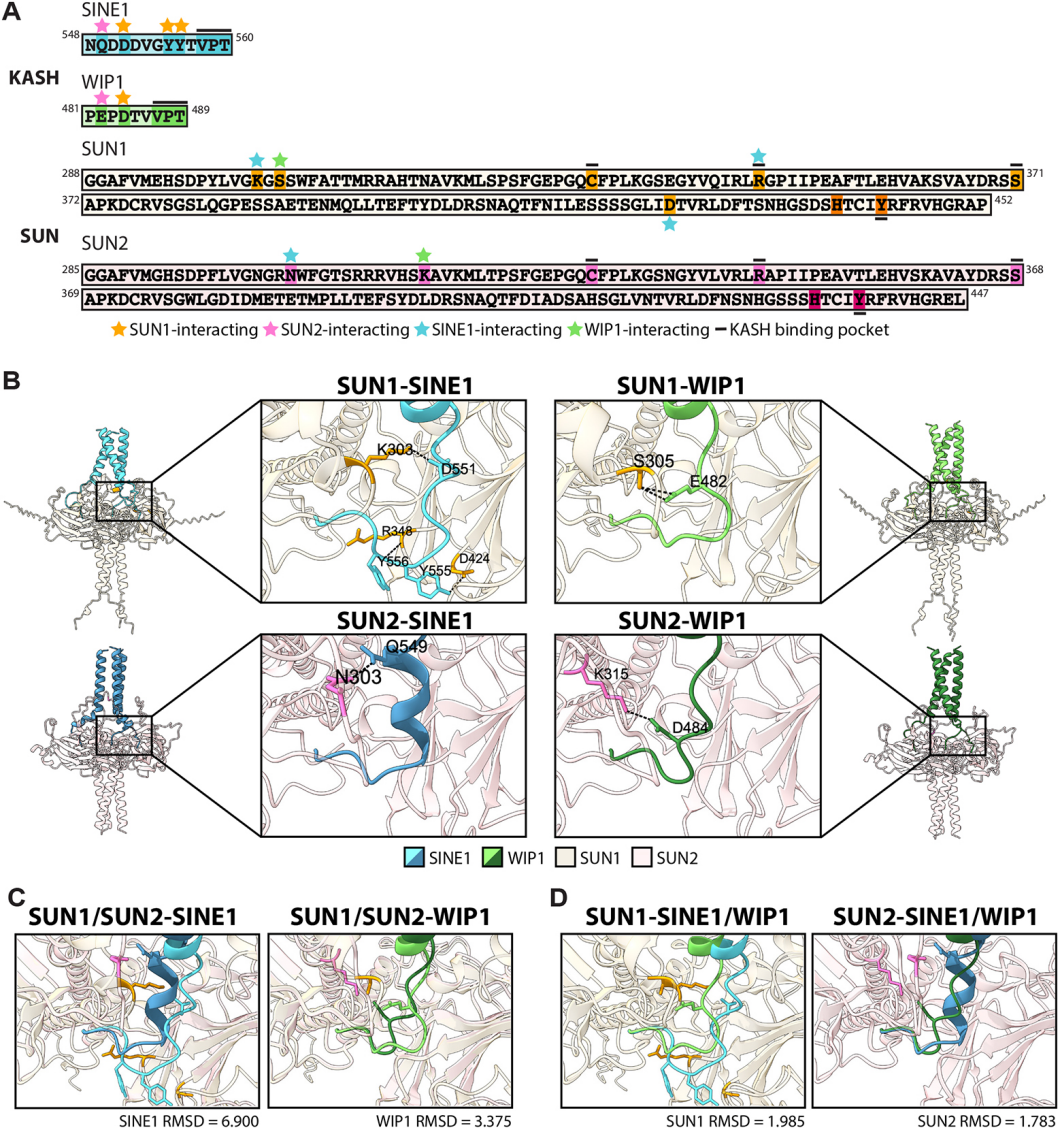
**Molecular modeling of SUN–KASH interaction.** (A) SINE1 and WIP1 KASH domain sequences and SUN1 and SUN2 SUN domain sequences. Highlighted residues indicate they are important to binding. Stars denote what interaction each residue is required for: SUN1 H-bond (orange), SUN2 H-bond (pink), SINE1 H-bond (turquoise) and WIP1 H-bond (green). Black line indicates conserved interactions in the KASH-binding pocket. Darker highlighted residues are previously reported KASH-binding pocket residues. (B) SUN1–SINE1, SUN1–WIP1, SUN2–SINE1 and SUN2–WIP1 predicted structures. Black dashed lines and highlighted residues (light orange or light pink) indicate unique H-bonding to specified SUN-KASH pair from Table S2. (C) Left, overlay of SUN1–SINE1 and SUN2–SINE1. Right, overlay of SUN1–WIP1 and SUN2–WIP1. RMSD shown compares the same KASH domain bound to different SUN proteins. (D) Left, overlay of SUN1–SINE1 and SUN1–WIP1. Right, overlay of SUN2–SINE1 and SUN2–WIP1. RMSD shown compares the same SUN protein bound to different KASH proteins. Each SUN–KASH model includes three KASH protein copies and three SUN protein copies. Structures were predicted using AlphaFold and visualized and annotated using ChimeraX.

With this confirmation in hand, we proceeded to interpret further KASH domain interactions between *Arabidopsis* SUN1 and SUN2. Each SUN–KASH pair had unique H-bonds that positioned the KASH domains slightly or very differently in the KASH-binding pocket (Fig. 6A,B; Table S2). Y555 and Y556 of SINE1 bound to D242 and R348 of SUN1, causing SINE1 to stick out into a distinct region of the KASH-binding pocket that was different from that seen in all other structures (Fig. 6B, top left). When overlaying structures of SINE1 bound to different SUN proteins (SUN1 or SUN2), SINE1 adopts two distinct SUN-binding modes (RMSD=6.900) with moderately strong H-bonding [SUN1, 2.782 Å, 2.808 Å, 2.932 Å, SUN2, 2.654 Å (1 Å=0.1 nm)], while overlays of WIP1 show less distinct binding modes (RMSD=3.375) and have weaker H-bonding (SUN1, 3.252 Å, 3.337 Å and 3.412 Å; SUN2, 3.404 Å) (Fig. 6C; Table S2). Upon overlaying the SUN1-binding structures for SINE1 and WIP1, we again saw how SINE1 adopted this distinct shape, whereas overlaying SINE1 and WIP1 bound to SUN2 gave very similar structures (Fig. 6D). Together, these modeling data suggest that KASH-binding pockets are highly structurally conserved across both *Arabidopsis* and humans, and that there is structural specificity between the binding modes of different plant KASH domains to different SUN proteins.

## DISCUSSION

In opisthokonts, LINC complexes are required for nuclear movement, nuclear positioning and nuclear anchorage (Starr and Fridolfsson, 2010). It has become increasingly apparent that LINC complexes are not ‘one size fits all’, but rather the components change dependent on the cellular or tissue context (King, 2023). The combination of SUN and KASH proteins at the core of the LINC complex are a platform for a wide variety of nucleoskeleton and cytoskeleton interactions. LINC complexes have become a popular model to study mechanotransduction due to the key role they play in connecting forces between the cytoskeleton and nucleus (King, 2023; Lele et al., 2018).

The multiplicity in function of LINC complexes is due to substantial differences in the cytoplasmic domains of KASH proteins, whereas the domains of LINC complexes that reside in the NE lumen are mostly thought to provide stability for the complex (Agrawal et al., 2022; Garner et al., 2023; Yu et al., 2011; Zhang et al., 2007). However, there is emerging evidence that the specificity of LINC complex function can also depend on differences in the SUN–KASH binding structure. In mammalian cells, it has been shown that the SUN domain plays a role in determining which cytoskeletal partner is recruited to interact with nesprin-2G and mediate nuclear positioning (Li et al., 2025; Zhu et al., 2017). The specificity of the SUN domain is intriguing given the high conservation across these domains, pointing to the importance of the perinuclear SUN-domain–KASH-domain interaction. Molecular dynamics simulations have shown that the human SUN2–KASH2 interaction is stable under a range of force amounts, and that this stability depends on paired cysteine residues in KASH2 and SUN2 (Jahed et al., 2015). In *C. elegans*, the KASH protein UNC-83 plays a role in nuclear migration, whereas ANC-1 is required for nuclear anchorage (Bone et al., 2014; Starr and Han, 2002). It has been shown that perturbing the structure of the KASH domain, including shortening, lengthening or swapping KASH domains, can diminish or abolish function, indicating the importance of the length and amino acid composition of the KASH domain (Cain et al., 2018; Jahed et al., 2015).

Plant KASH domains are substantially shorter than animal KASH domains and do not contain cysteine residues, and there is variability in both length and structure across plant KASH domains. Prior investigation of required components of the plant KASH domain included deletion of the terminal four amino acids, which abolishes the interaction with SUNs (Newman-Griffis et al., 2019; Zhou et al., 2012, 2014). The chimeric KASH proteins employed here swapped the entire KASH domain, changing length, amino acid composition and structure of the domain.

Here, we have reported that the chimeric WIP1^SINE1KASH^ has a variable ability to replace WIP1 in different cellular roles. In trichoblasts, WIP1^SINE1KASH^ is unable to complement the *wip123* nuclear shape and nuclear size defects (Fig. 3C,D). In pollen tubes, WIP1^SINE1KASH^ is able to complement the MGU order defect in *wip123* (Fig. 4B,C). One possible explanation for this difference is the role SUN proteins play. In mature root hairs, although both SUN proteins are expressed in root hairs, SUN1 is much more highly expressed than SUN2 in all developmental stages, including trichoblasts (Fig. S3A; Shahan et al., 2022). Consistent with this expression data, it has also been shown that SUN1 is required for WT-like nuclear shape and size, whereas SUN2 is dispensable for this function (Oda and Fukuda, 2011; Zhou et al., 2015a). In pollen tubes, where SUN1 and SUN2 expression are more similar (Fig. S3B right; Guo et al., 2025; Nam et al., 2026), SUN1 and SUN2 are functionally redundant for MGU migration (Zhou et al., 2015b). Interestingly, our modeling suggests that the SUN1–SINE1 binding mode differs significantly from that seen for SUN1–WIP1, whereas this is not the case for SUN2 (Fig. 6D, left). Therefore, the difference in SUN1–SINE1 binding structure and strength, compared to that of SUN1–WIP1, could explain the inability of WIP1^SINE1KASH^ to replace WIP1 in trichoblasts and complement the *wip123* phenotype. In pollen, where SUN2 alone has been shown to be sufficient to maintain MGU migration order, differences in the SUN1–SINE1 binding structure could be compensated for by SUN2–SINE1, which is very similar to SUN2–WIP1 in our modeling (Fig. 6D, right). This could explain the ability of WIP1^SINE1KASH^ to complement the *wip123* MGU order defect in pollen tubes.

A variety of motor proteins have been identified that interact with the SUN–WIP–WIT LINC complex. In root hairs, Myosin XI-I interacts with WIPs and WIT2, and is required for nuclear shape and nuclear movement (Tamura et al., 2013; Zhou et al., 2015a). The pollen-expressed kinesin-14 proteins HUG1 and HUG2 have recently been shown to interact with WIPs and WITs (Chang et al., 2025; Yan et al., 2025). In addition to their localization to the VN, these kinesins localize to the surface of the sperm cells (SCs) and appear to be required for the connection between the VN and SCs (Chang et al., 2025). The difference in motor proteins bound by the SUN–WIP–WIT LINC complex indicates a possibility that the force required for distinct SUN–KASH–motor interactions could vary between the two cell types.

In guard cells, it has been established that SINE1 is required for reorganization of the actin and microtubule cytoskeleton during ABA-induced stomatal closure (Biel et al., 2020a,b, 2022). To this point, no SINE1-interacting cytoplasmic proteins have been identified, and the mode of action for SINE1 in cytoskeletal rearrangement has not been identified. The chimeric KASH protein SINE1^WIP1KASH^ fails to complement the *sine1-1* stomatal closure defect, indicating a specificity for the SINE1 KASH domain in this process (Fig. 2B–D). Our modeling suggests that the SINE1 KASH domain, which is longer than the WIP1 KASH domain, undergoes more hydrogen bonding with SUN1 compared with WIP1 (Fig. 6; Table S2). Although SUN1 is slightly more highly expressed than SUN2, the role of SUN proteins in stomatal closure has yet to be established (Fig. S3B, left; Guo et al., 2025; Nam et al., 2026). This could indicate that the length of the KASH domain, and the ability to form additional hydrogen bonds, is required for the role SINE1 plays in cytoskeletal rearrangement during stomatal closure.

Here, we have modeled the KASH-binding pocket of two *Arabidopsis* SUN proteins bound to two distinct KASH proteins (Fig. 5). Plant SUN proteins were initially identified through homology to opisthokont SUN proteins (Graumann et al., 2010; Murphy et al., 2010). However, the C-terminus of plant SUNs, including the SUN domain, has significant divergence from animal SUNs (Zhou and Meier, 2013). Despite the sequence differences between plant and animal SUNs, the SUN–KASH binding pocket is highly conserved (Fig. 5D). This is consistent with previously published data showing that mutations of the conserved histidine (H349 or H434) and tyrosine residues (Y443 or Y438) deep within the binding pocket disrupt KASH binding (Fig. 5A) (Newman-Griffis et al., 2019; Zhou et al., 2014). These SUN mutants have also been shown to phenocopy known plant KASH mutant defects (Newman-Griffis et al., 2019; Zhou and Meier, 2013; Zhou et al., 2014, 2015b). Additionally, although the multimeric structure of *Arabidopsis* LINC complexes has not been fully elucidated, the modeling presented here suggests plant SUNs form a trimeric complex that can bind three separate KASH domains, similar to what is found for opisthokont SUNs (Fig. S2C) (Sosa et al., 2012). Taken together, these SUN–KASH models provide a new avenue to understand the differences between opisthokont and plant LINC complexes.

The data shown in this study provide further evidence that SUN–KASH pairs are specialized for cellular functions. This is reminiscent of the work done in *C. elegans*, where the KASH domains of UNC-83 and ANC-1 are differentially required for functions in nuclear movement and nuclear anchorage (Cain et al., 2018; Jahed et al., 2019). The plant KASH proteins studied here lack many of the features identified in opisthokont KASH proteins as being important to LINC complex integrity, including cysteine residues. Despite this, the differences in WIP1 and SINE1 shown here indicate that length and structure remain important variables in ‘customizing’ SUN–KASH interactions for function in plants. We now have tools in hand that could be used to further examine this specificity for LINC complex function in plants. Prior data have shown from co-immunoprecipitation in overexpressing tissues that WIP1–SUN1, WIP1–SUN2, SINE1–SUN1 and SINE1–SUN2 all interact. The SUN–KASH interactions depend on the last four amino acids of the respective KASH domains and the two conserved amino acids in the SUN domains, that our models implicate in binding (Zhou et al., 2012, 2014, 2015b). The application of more sophisticated, quantitative *in vitro* protein-binding assays to determine binding constants might be able to resolve the differences in *in vivo* functional interactions we have reported here. In addition, future work could use site-directed mutagenesis to perturb the important hydrogen bonding residues that our models propose, to test their requirement for the specific *in vivo* LINC complex functions. Investigation of mechanotransduction across the plant LINC complexes is an area that is largely unexplored. Measuring the *in vivo* forces experienced between different plant LINC complexes could shed light on how plant LINC complexes facilitate different cellular functions.

## MATERIALS AND METHODS

### Plant material

*Arabidopsis thaliana* seeds (Columbia-0, Col-0, ecotype) were germinated and grown on Murashige and Skoog (MS) agar (0.8%) plates (Caisson Laboratories, Smithfield, UT, USA), under constant white light of ∼100 PAR (μmol/m^2^/s) at 25°C. Seedlings at the four-leaf stage were transplanted into soil and grown under long-day conditions (16 h light, 8 h dark), unless otherwise stated. Col-0 *Arabidopsis* seeds were obtained from the *Arabidopsis* Biological Resource Center (ABRC, Columbus, OH, USA). The *wip1-1* (SAIL_390_A08) *wip2-1* (SALK_052226) *wip3-1* (GABI-Kat 459H07) (‘*wip123’*) mutant and the *sine1-1* (SALK_018239C) mutant were previously reported (Xu et al., 2007; Zhou et al., 2014). WIP1_pro_::GFP-WIP1 in *wip123* and SINE1_pro_::GFP-SINE1 in *sine1-1* have been previously described (Zhou and Meier, 2014; Zhou et al., 2014). Heterozygous *male sterility-1* (*ms-1*) was obtained from the ABRC (Yang et al., 2007).

### Cloning KASH domain-swap chimeras

Coding sequences for SINE1^WIP1KASH^ and WIP1^SINE1KASH^ were amplified using reverse primers that anneal upstream of the native KASH domains with overhangs consisting of the coding sequence for the replacement KASH domains, using SINE1 and WIP1 cDNAs as a template, and cloned into pENTR/D-Topo (Invitrogen) (Table S1). Following sequence confirmation, both SINE1^WIP1KASH^ and WIP1^SINE1KASH^ were recombined into N-terminal GFP fusion vectors, to generate GFP–SINE1^WIP1KASH^ and GFP–WIP1^SINE1KASH^, using LR Clonase II (Invitrogen, Carlsbad, CA, USA). The chimeric KASH sequences were recombined into either the plant expression vector pH7WGF2 (for transient *N. benthamiana* infiltrations; Karimi et al., 2002), or pHSINE1proAG (Zhou et al., 2014) and pHWIP1pro2200 (Zhou and Meier, 2014) (for generation of transgenic *Arabidopsis* lines).

### *Agrobacterium* transformation

*Agrobacterium tumefaciens* (strain ABI) was transformed via triparental mating as described previously (Wise et al., 2006). In short, *E. coli* carrying the construct of interest was co-incubated on lysogeny broth (LB) agar (1.5%) plates with ABI and *E. coli* helper strain pRK2013 at 30°C overnight. Colonies were then streaked on LB plates containing appropriate antibiotics to select for transformed ABI, and size was confirmed by PCR.

### *N. benthamiana* infiltrations

Leaves of 3-week-old non-flowering *N. benthamiana* plants were transiently co-infiltrated with ABI, containing pH7WGF2 constructs and histone 2B–mCherry (H2B–mCherry), as a nuclear marker (Newman-Griffis et al., 2019). Bacteria were precipitated then resuspended in 10 mM MES pH 5.6, 10 mM MgCl_2_ and 100 μM acetosyringone to an optical density at 600 nm (OD_600_)<1.0 (Avisar et al., 2009). *N. benthamiana* leaves were pressure infiltrated with ABI with a plastic syringe. After 3 days, infiltrated leaves were imaged via confocal microscopy.

### Confocal microscopy

Confocal microscopy was performed using a Nikon Eclipse C2plus confocal laser microscope. Samples were excited with a wavelength of 488 nm and the emission was detected at 516 nm. Laser levels were maintained consistently across similar samples with the GFP laser set to 3–4 with a gain of 40–60. Different objectives were used for different tissues: 60× oil (*N. benthamiana* infiltrations, *Arabidopsis* root tips), 40× water (stomata), 20× air (trichoblasts, pollen tubes).

### *Arabidopsis* stable transformation

Stable transgenic *Arabidopsis* lines were generated via ABI-mediated floral transformation as described previously (Clough and Bent, 1998). LB liquid medium was inoculated with ABI containing constructs of interest and were grown overnight at 30°C. Bacteria were collected by centrifugation (1750 ***g*** for 15 min) and resuspended in 5% sucrose in H_2_O and 300 ml/l silwet L-77 to OD_600_=0.8. Meristematic regions (inflorescence and branching points) of *sine1-1* and *wip123* plants with many bolts were dipped in the bacterial suspension. Plants were laid horizontally and kept moist overnight at room temperature, then moved to a growth chamber to allow seeds to set. T_1_ transgenics were selected on MS agar (0.8%) plates with 30 μg/ml hygromycin (Sigma-Aldrich, St. Louis, MO, USA) and screened for GFP fluorescence via confocal microscopy. From each set of confirmed transformants expressing GFP, we chose one line with an equal or higher expression level (GFP–SINE1^WIP1KASH^#8 and GFP–WIP1^SINE1KASH^#5) than the comparison line GFP–WIP1 in *wip123* or GFP–SINE1 in *sine1-1*, and one line with a lower expression level (GFP–SINE1^WIP1KASH^#7 and GFP–WIP1^SINE1KASH^#4). Because both WIP1 and SINE1 are expressed in seedling root tips, we used this tissue for comparing GFP expression levels (Fig. 1E). GFP–SINE1^WIP1KASH^#8, GFP–WIP1^SINE1KASH^#5, GFP–SINE1^WIP1KASH^#7 and GFP–WIP1^SINE1KASH^#4 were deposited at the ABRC (Columbus, OH, USA).

### Stomatal aperture measurements

Stomatal assays were performed as previously described (Biel et al., 2020a). In short, leaves of 5–7-week-old plants, grown under short-day conditions (8 h light, 16 h dark; white light of ∼100 PAR), were detached and placed in a Petri dish abaxial side up in opening buffer (OB; 10 mM of MES pH 6.15, 20 µM CaCl_2_ and 50 mM KCl) for 3 h of constant light (Biel et al., 2020a; Li et al., 2014; Zhao et al., 2011). At designated timepoints, epidermal peels were mounted in OB and imaged using confocal microscopy. For stomatal closing assays, the 3 h light in OB was followed by 3 h dark in closing buffer [CB; 10 mM MES pH 6.15, 20 µM ABA (Sigma-Aldrich, St. Louis, MO, USA)] (Biel et al., 2020a; Zhao et al., 2011). The stomatal pore, defined between darkest edges, was measured using FIJI software (https://imagej.net/software/fiji/).

### Trichoblast nuclear shape measurement

7-day-old seedlings were grown on MS agar (0.8%) plates. Whole seedlings were stained by a 1:4000 dilution of 10,000× SYBR green concentrate (Thermo Fisher Scientific, Waltham, MA, USA) in 0.5× MS and 1% sucrose for 20 min, destained for 20 min, then imaged using confocal microscopy. Trichoblasts were chosen in which the nucleus had not emerged into the tip, but the beginning of a bulge was visible. Circularity of the nucleus was quantified by tracing the outside of the nucleus and measuring in FIJI using the equation 4π×area/perimeter^2^.

### Semi-*in vivo* pollen germination assay

Stigmas of *male sterility 1* (*ms1*) plants (Yang et al., 2007) were saturated with pollen of interest from fully open flowers of 4–5-week-old plants, as described previously (Moser et al., 2020). After 2 h in light, stigma were dissected and placed horizontally onto 0.4% agarose pads containing pollen germination medium [PGM; 5 mM KCl, 5 mM CaCl_2_, 1 mM Ca(NO_3_)_2_, 1 mM MgSO_4_, 10% sucrose, 0.01% boric acid, pH 7.5] on a glass slide, suspended in liquid PGM containing a 1:4000 dilution of 10,000× SYBR green concentrate (Thermo Fisher Scientific) (Mendes et al., 2025; Motomura et al., 2021) and placed in a humidity chamber for 3 h (Moser et al., 2020; Zhou and Meier, 2014). After 3 h of pollen tube germination (5 h post pollination) pollen tubes were imaged using confocal microscopy.

### Molecular modeling

*Arabidopsis* SUN and KASH protein sequences were obtained from UniProt (https://www.uniprot.org/; SINE1, Q5XVI1; WIP1, Q8GXA4; SUN1, Q9FF75; SUN2, Q9SG79) (The UniProt Consortium, 2019). Human SUN-KASH structures were downloaded from the Protein Data Bank (PDB, https://www.rcsb.org/; KASH1–SUN2, PDB code 4DXR; KASH4–SUN2, PDB code 6WMD) (Berman, 2000; Burley et al., 2025). Domains were defined using UniProt and AlphaFold 3. The AlphaFold3 AlphaFold Server was used to predict SUN–KASH binding of *Arabidopsis* SUN and KASH proteins and for ipTM confidence measures (Abramson et al., 2024; Jumper et al., 2021). ChimeraX was used to visualize results, calculate RMSD values and calculate H-bonds (Pettersen et al., 2021). Intramodel H-bonding residues that were identified were selected and highlighted using ChimeraX, with a distance tolerance of 0.400 Å and angle tolerance of 20.000°.

### Statistical analysis

Error bars represent mean±s.d. Letters represent statistical groups (*P*<0.05) calculated using a two-way ANOVA (Table S3) or one-way ANOVA (Table S4) compared within each timepoint, paired with Tukey's honestly significant difference test. Statistics were calculated in R software using base stats and the emmeans package (https://rvlenth.github.io/emmeans/). For MGU order statistics, a two-tailed Fisher's exact test was used, where **P<0.01 and ^O^*P*>0.05, when compared with the WT.

## Supplementary Material

10.1242/joces.264672_sup1 Supplementary information
