# Supplementary material for: Arabidopsis KASH domains are differentially required for distinct LINC complex roles in stomata, roots and pollen
Source: J Cell Sci. 2026 Apr 30;139(12):jcs264672. doi: 10.1242/jcs.264672 (PMC13200780; doi:10.1242/jcs.264672)
Supplement: Supplementary information [file joces-139-264672-s1.pdf]

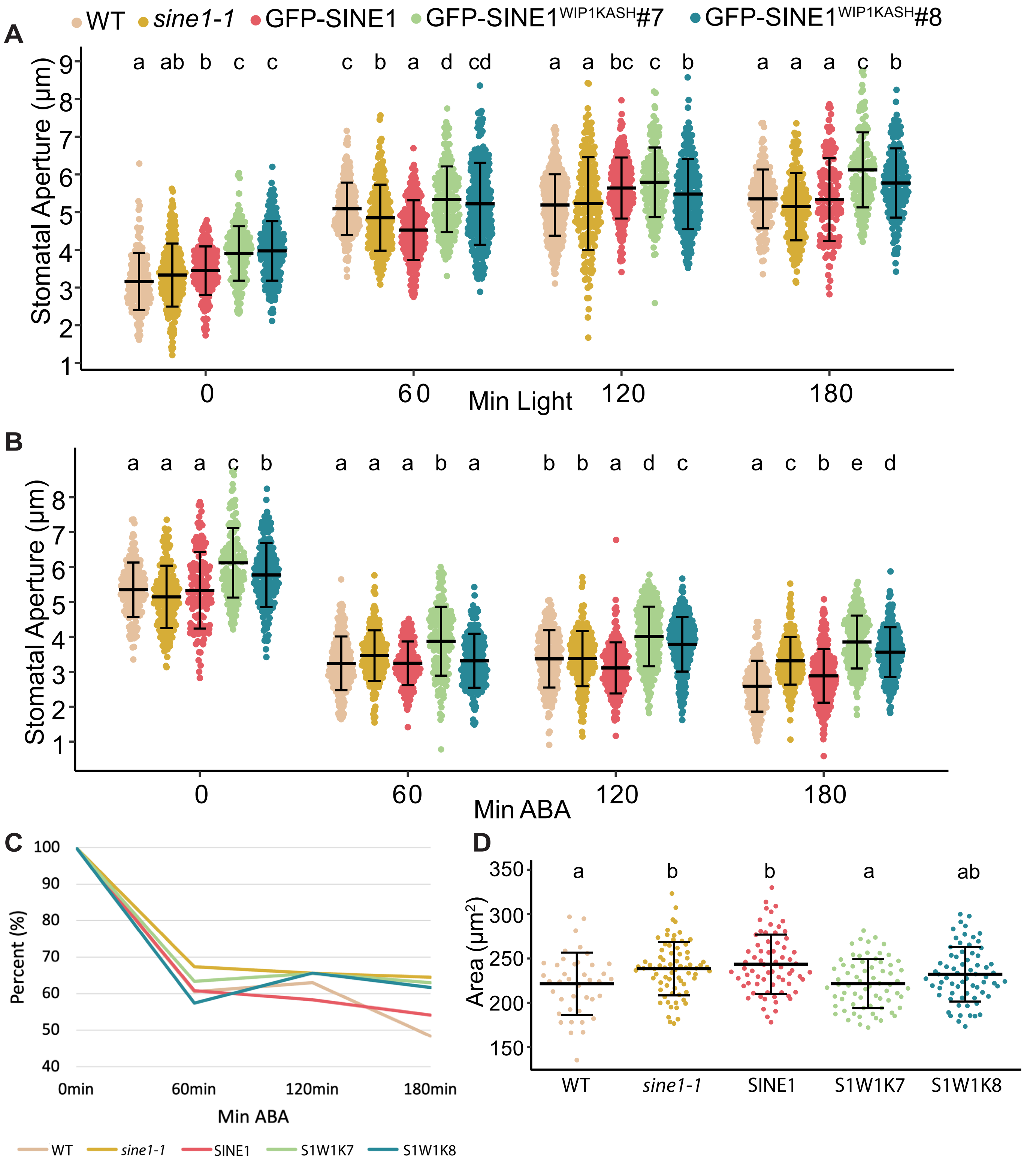

Fig. S1. The SINE1 KASH domain is required for stomatal dynamics throughout stomatal closure. Leaves from 5-6 week old short day plants were placed in **(A)** opening buffer containing Ca^{2+} and K^{+} for 180 min then **(B)** transferred to closing buffer containing 20 µM ABA over 180 min of darkness. N>130 for each line, 3 biological repeats. Two-way ANOVA, p<0.05, paired with Tukey's HSD, letters represent statistical groups, bars mean ± SD (Table S3). **(C)** Percent of open stomatal pore size confirms stomatal closure defect of SINE1^{WIP1KASH}. 0 min ABA was used as 100 % for each genotype; 180 min ABA was reported as a percentage of the 0 min pore measurement. S1W1K, SINE1^{WIP1KASH}. **(D)** Stomatal area is not impacted by the WIP1 KASH domain. Measurements were made on the 180 min timepoint of one biological replicate of the ABA induced stomatal assay in Figure 3D. N>50 for each line. One-way ANOVA, p<0.05, paired with Tukey's HSD, letters represent statistical groups, bars mean ± SD (Table S3).

Journal of Cell Science • Supplementary information

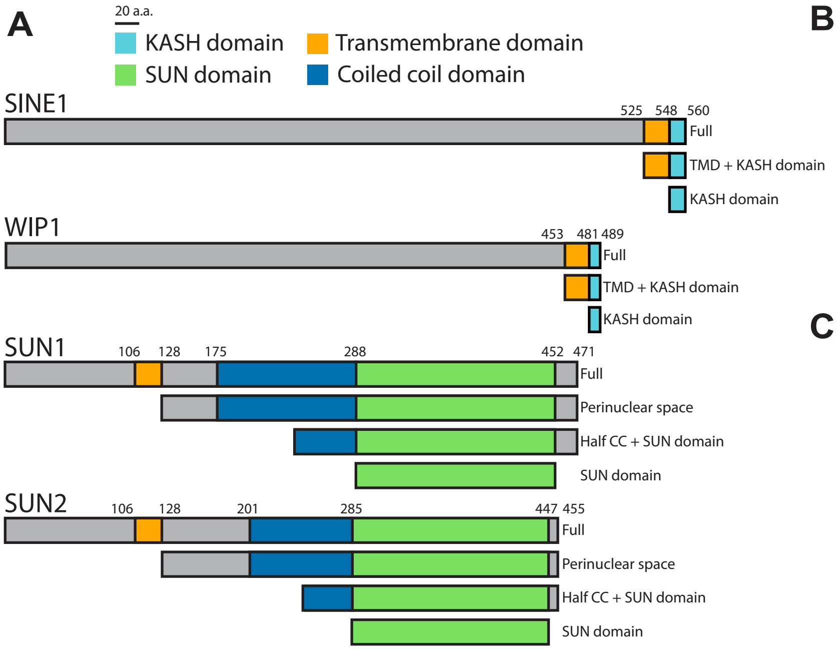

B

Domain condition	SUN1-SINE1	SUN1-WIP1	SUN2-SINE1	SUN2-WIP1
**KASH:** Full<br>**SUN:** Full	0.32	0.36	0.34	0.34
**KASH:** TMD + KASH domain<br>**SUN:** Perinuclear space	0.59	0.59	0.54	0.57
**KASH:** TMD + KASH domain<br>**SUN:** Half CC + SUN domain	0.76	0.76	0.78	0.79
**KASH:** KASH domain<br>**SUN:** SUN domain	0.20	0.66	0.7	0.73

C

Multimers	SUN1-SINE1	SUN1-WIP1	SUN2-SINE1	SUN2-WIP1
1	0.56	0.25	0.26	0.65
2	0.31	0.43	0.57	0.14
3	0.76	0.76	0.78	0.79
4	0.53	0.51	0.18	0.39
5	0.46	0.46	0.49	0.46
6	0.45	0.44	0.44	0.44

Fig. S2. Determining modeling conditions. (A) Gene diagram of SINE1, WIP1, SUN1, and SUN2 truncations. Colored according to key above. **(B)** Selection of domains to model. Table reports ipTM values. All modelled with 3 copies of each SUN and KASH protein. Regions defined by domain descriptions in panel A. **(C)** Determining preferred multimer state. Table reports ipTM values. Red indicates ipTM value below 0.6 for failed predictions.

Journal of Cell Science • Supplementary information

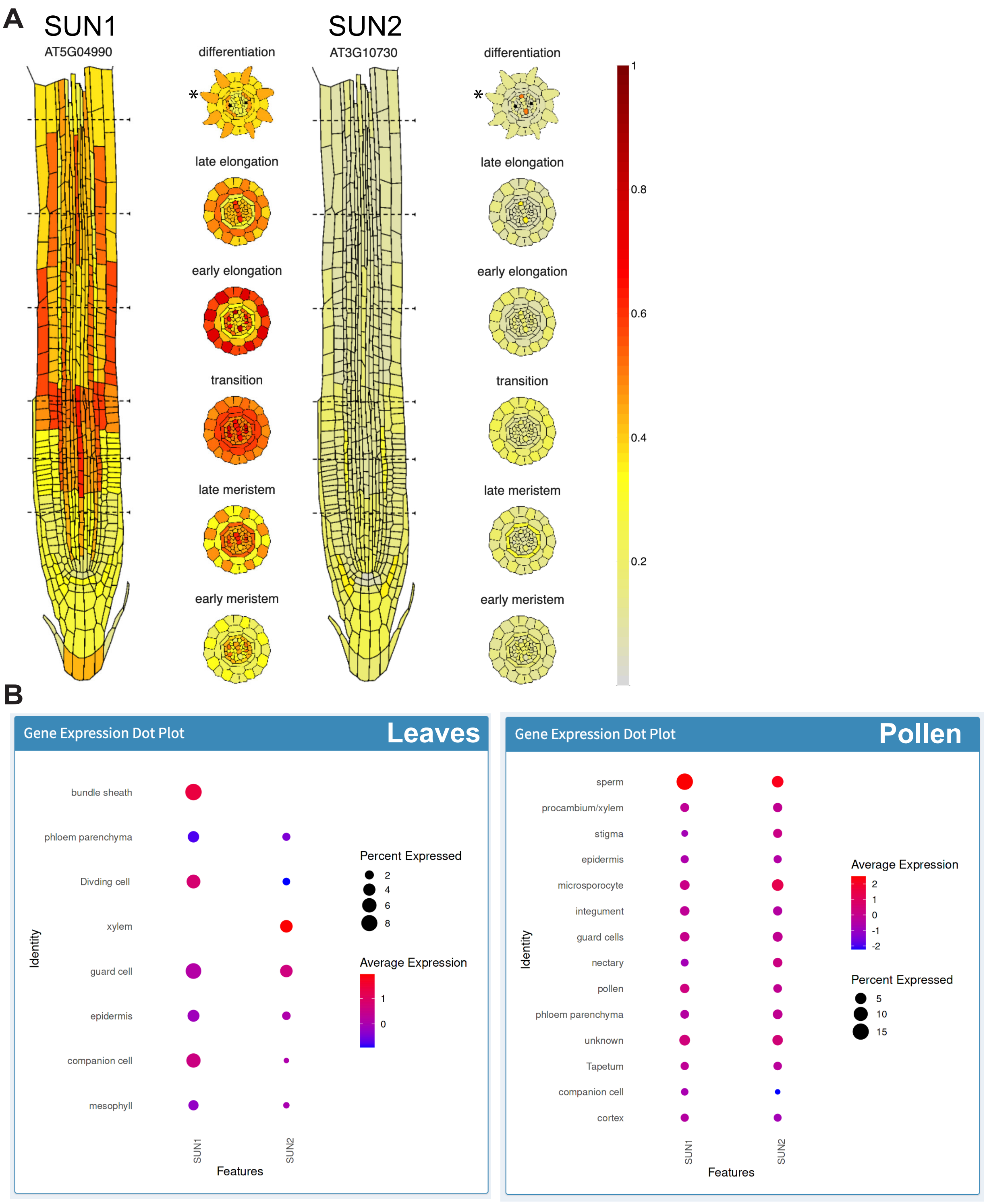

Fig. S3. Expression levels of SUN1 and SUN2. (A) SUN1 and SUN 2 expression in roots (Shahan et al., 2022; https://rootcellatlas.org). * indicates trichoblasts. **(B)** SUN1 and SUN2 expression in leaves and flowers (Guo et al., 2025; Nam et al., 2026; https://kdhulipalla13-gene-co-expression-2-0.share.connect.posit.cloud/).

Journal of Cell Science • Supplementary information

Table S1. Primers used in this study. SINE1F and SINE1WIP1KASH R were used to clone SINE1^{WIP1KASH}. WIP1F and WIP1SINE1KASH R were used to clone WIP1^{SINE1KASH}. Lowercase indicates overhang which does not anneal to either SINE1 or WIP1 coding sequence. In SINE1F and WIP1F “cacc" is included for directional cloning. Lowercase portions in SINE1WIP1KASH R and WIP1SINE1KASH R correspond to the opposite KASH domain.

Name	Sequence (5’-3’)
SINE1F	caccATGGGTTTGAATCTGAATCCAATAT
WIP1F	caccATGGATTTGGAGAGTGAAAGCTCTGCAC
SINE1WIP1 KASH R	tcatgtgggtacaacagtatctggctccggCACCATCAATATCACAGTTGCGAA
WIP1SINE1 KASH R	tcatgtagggacagtgtagtaaccaacatcatcatcttgattcAACAATTGAGACAGGAGCAACAAAACTAC

Journal of Cell Science • Supplementary information

Table S2. SUN-KASH predicted H-bonds. H-bonds were determined and donor-acceptor distances calculated using ChimeraX. H-bonds that are conserved across SUN-KASH structures are highlighted in green. H-bonds that are conserved across SUN-WIP1 structures are highlighted in yellow. H-bonds that are unique to a specific SUN-KASH pair are highlighted in orange. H-bonding atoms are identified using ChimeraX nomenclature. D-A indicates donor-acceptor distance.

SUN1-SINE1

Donor	Atom	Acceptor	Atom	D-A (Å)
SUN1A K303	NZ	SINE1 D551	OD1	2.782
SUN1A C333	SG	SINE1 P559	O	3.828
SUN1A S371	OG	SINE1 T560	O	2.686
SUN1A S371	OG	SINE1 T560	OXT	3.277
SUN1A Y443	OH	SINE1 T560	OXT	2.74
SUN1A R348	NH1	SINE1 V558	O	2.836
SINE1 Y555	OH	SUN1C D424	OD2	2.808
SINE1 Y556	N	SUN1C R348	O	2.932

SUN1-WIP1

Donor	Atom	Acceptor	Atom	D-A (Å)
SUN1A S305	OG	WIP1 E482	OE1	3.252
SUN1A S305	OG	WIP1 E482	OE2	3.337
SUN1A C333	SG	WIP1 P488	O	3.763
SUN1A S371	OG	WIP1 T489	O	2.492
SUN1A S371	OG	WIP1 T489	OXT	3.433
SUN1A Y443	OH	WIP1 T489	OXT	2.462
SUN1C R348	NH1	WIP1 V487	O	2.846
SUN1C R348	NH2	WIP1 V487	O	3.412

SUN2-SINE1

Donor	Atom	Acceptor	Atom	D-A (Å)
SUN2A C330	SG	SINE1 P559	O	3.855
SUN2A S368	OG	SINE1 T560	O	2.537
SUN2A S368	OG	SINE1 T560	OXT	3.431
SUN2A Y438	OH	SINE1 T560	OXT	2.521
SUN2C R345	NH1	SINE1 V558	O	2.942
SINE1 Q549	NE2	SUN2A N303	OD1	2.654

SUN2-WIP1

Donor	Atom	Acceptor	Atom	D-A (Å)
SUN2A K315	NZ	WIP1 D484	OD2	3.404
SUN2A C330	SG	WIP1 P488	O	3.835
SUN2A S368	OG	WIP1 T489	O	2.648
SUN2A S368	OG	WIP1 T489	OXT	3.399
SUN2A Y438	OH	WIP1 T489	OXT	2.675
SUN2C R345	NH1	WIP1 V487	O	2.846
SUN2C R345	NH2	WIP1 V487	O	3.400

Table S3A

Lines	Min	p-value
(sine1-1) - WT	0	1
(GFP-SINE1) - WT	0	3.1E-01
(GFP-SINE1) - (sine1-1)	0	1
(GFP-SINE1WIP1KASH #7) - WT	0	3.1E-12
(GFP-SINE1WIP1KASH #7) - (sine1-1)	0	6.0E-09
(GFP-SINE1WIP1KASH #7) - (GFP-SINE1)	0	1.5E-04
(GFP-SINE1WIP1KASH #8) - WT	0	1.7E-14
(GFP-SINE1WIP1KASH #8) - (sine1-1)	0	3.8E-11
(GFP-SINE1WIP1KASH #8) - (GFP-SINE1)	0	4.4E-06
(GFP-SINE1WIP1KASH #8) - (GFP-SINE1WIP1KASH #7)	0	1
(sine1-1) - WT	60	4.3E-01
(GFP-SINE1) - WT	60	4.8E-09
(GFP-SINE1) - (sine1-1)	60	2.3E-02
(GFP-SINE1WIP1KASH #7) - WT	60	3.6E-01
(GFP-SINE1WIP1KASH #7) - (sine1-1)	60	1.4E-05
(GFP-SINE1WIP1KASH #7) - (GFP-SINE1)	60	2.4E-16
(GFP-SINE1WIP1KASH #8) - WT	60	1
(GFP-SINE1WIP1KASH #8) - (sine1-1)	60	1.1E-03
(GFP-SINE1WIP1KASH #8) - (GFP-SINE1)	60	2.4E-14
(GFP-SINE1WIP1KASH #8) - (GFP-SINE1WIP1KASH #7)	60	1
(sine1-1) - WT	120	1
(GFP-SINE1) - WT	120	5.1E-07
(GFP-SINE1) - (sine1-1)	120	2.5E-04
(GFP-SINE1WIP1KASH #7) - WT	120	3.4E-10
(GFP-SINE1WIP1KASH #7) - (sine1-1)	120	4.7E-07
(GFP-SINE1WIP1KASH #7) - (GFP-SINE1)	120	1
(GFP-SINE1WIP1KASH #8) - WT	120	2.6E-02
(GFP-SINE1WIP1KASH #8) - (sine1-1)	120	4.3E-01
(GFP-SINE1WIP1KASH #8) - (GFP-SINE1)	120	1
(GFP-SINE1WIP1KASH #8) - (GFP-SINE1WIP1KASH #7)	120	9.1E-02
(sine1-1) - WT	180	1
(GFP-SINE1) - WT	180	1
(GFP-SINE1) - (sine1-1)	180	1
(GFP-SINE1WIP1KASH #7) - WT	180	1.2E-11
(GFP-SINE1WIP1KASH #7) - (sine1-1)	180	3.0E-19
(GFP-SINE1WIP1KASH #7) - (GFP-SINE1)	180	2.7E-11
(GFP-SINE1WIP1KASH #8) - WT	180	2.1E-03
(GFP-SINE1WIP1KASH #8) - (sine1-1)	180	2.5E-08
(GFP-SINE1WIP1KASH #8) - (GFP-SINE1)	180	2.1E-03
(GFP-SINE1WIP1KASH #8) - (GFP-SINE1WIP1KASH #7)	180	4.5E-02

Journal of Cell Science • Supplementary information

Table S3B

Line	Min	emmean	SE	df	LCL	UCL	group
WT	0	3.16107	0.072	3671	3.020	3.303	a
sine1-1	0	3.33098	0.058	3671	3.218	3.444	ab
GFP-SINE1	0	3.44681	0.070	3671	3.310	3.584	b
GFP-SINE1WIP1KASH #7	0	3.90312	0.066	3671	3.773	4.033	c
GFP-SINE1WIP1KASH #8	0	3.97155	0.067	3671	3.840	4.103	c
GFP-SINE1	60	4.52344	0.065	3671	4.397	4.650	a
sine1-1	60	4.85256	0.065	3671	4.726	4.980	b
WT	60	5.09061	0.058	3671	4.977	5.204	c
GFP-SINE1WIP1KASH #8	60	5.22098	0.055	3671	5.112	5.330	cd
GFP-SINE1WIP1KASH #7	60	5.33896	0.067	3671	5.207	5.471	d
WT	120	5.18871	0.049	3671	5.092	5.285	a
sine1-1	120	5.22610	0.066	3671	5.097	5.355	a
GFP-SINE1WIP1KASH #8	120	5.47913	0.065	3671	5.352	5.606	b
GFP-SINE1	120	5.63947	0.060	3671	5.521	5.758	bc
GFP-SINE1WIP1KASH #7	120	5.78986	0.071	3671	5.650	5.930	c
sine1-1	180	5.14590	0.071	3671	5.006	5.285	a
GFP-SINE1	180	5.33386	0.078	3671	5.180	5.488	a
WT	180	5.35055	0.073	3671	5.207	5.494	a
GFP-SINE1WIP1KASH #8	180	5.77301	0.070	3671	5.636	5.910	b
GFP-SINE1WIP1KASH #7	180	6.12082	0.074	3671	5.975	6.266	c

Table S3C

Lines	Min	p-value
(sine1-1) - WT	0	1
(GFP-SINE1) - WT	0	1
(GFP-SINE1) - (sine1-1)	0	1
(GFP-SINE1WIP1KASH #7) - WT	0	7.4E-14
(GFP-SINE1WIP1KASH #7) - (sine1-1)	0	7.9E-23
(GFP-SINE1WIP1KASH #7) - (GFP-SINE1)	0	1.9E-13
(GFP-SINE1WIP1KASH #8) - WT	0	3.8E-04
(GFP-SINE1WIP1KASH #8) - (sine1-1)	0	6.0E-10
(GFP-SINE1WIP1KASH #8) - (GFP-SINE1)	0	3.8E-04
(GFP-SINE1WIP1KASH #8) - (GFP-SINE1WIP1KASH #7)	0	1.4E-02
(sine1-1) - WT	60	5.7E-01
(GFP-SINE1) - WT	60	1
(GFP-SINE1) - (sine1-1)	60	9.6E-01
(GFP-SINE1WIP1KASH #7) - WT	60	5.4E-11
(GFP-SINE1WIP1KASH #7) - (sine1-1)	60	2.1E-04
(GFP-SINE1WIP1KASH #7) - (GFP-SINE1)	60	1.0E-09
(GFP-SINE1WIP1KASH #8) - WT	60	1
(GFP-SINE1WIP1KASH #8) - (sine1-1)	60	1
(GFP-SINE1WIP1KASH #8) - (GFP-SINE1)	60	1
(GFP-SINE1WIP1KASH #8) - (GFP-SINE1WIP1KASH #7)	60	7.4E-08
(sine1-1) - WT	120	1
(GFP-SINE1) - WT	120	1.4E-01
(GFP-SINE1) - (sine1-1)	120	1.1E-01
(GFP-SINE1WIP1KASH #7) - WT	120	5.6E-13
(GFP-SINE1WIP1KASH #7) - (sine1-1)	120	8.8E-13
(GFP-SINE1WIP1KASH #7) - (GFP-SINE1)	120	1.6E-25
(GFP-SINE1WIP1KASH #8) - WT	120	1.0E-05
(GFP-SINE1WIP1KASH #8) - (sine1-1)	120	1.4E-05
(GFP-SINE1WIP1KASH #8) - (GFP-SINE1)	120	1.6E-15
(GFP-SINE1WIP1KASH #8) - (GFP-SINE1WIP1KASH #7)	120	2.7E-01
(sine1-1) - WT	180	4.6E-15
(GFP-SINE1) - WT	180	2.6E-02
(GFP-SINE1) - (sine1-1)	180	1.3E-06
(GFP-SINE1WIP1KASH #7) - WT	180	6.0E-43
(GFP-SINE1WIP1KASH #7) - (sine1-1)	180	5.4E-09
(GFP-SINE1WIP1KASH #7) - (GFP-SINE1)	180	4.0E-32
(GFP-SINE1WIP1KASH #8) - WT	180	1.9E-25
(GFP-SINE1WIP1KASH #8) - (sine1-1)	180	2.1E-01
(GFP-SINE1WIP1KASH #8) - (GFP-SINE1)	180	1.4E-15
(GFP-SINE1WIP1KASH #8) - (GFP-SINE1WIP1KASH #7)	180	4.4E-02

Journal of Cell Science • Supplementary information

Table S3D

Line	Min	emmean	SE	df	LCL	UCL	group
sine1-1	0	5.14590	0.065	3494	5.018	5.274	a
GFP-SINE1	0	5.33386	0.072	3494	5.193	5.475	a
WT	0	5.35055	0.067	3494	5.219	5.482	a
GFP-SINE1WIP1KASH #8	0	5.77301	0.064	3494	5.647	5.899	b
GFP-SINE1WIP1KASH #7	0	6.12082	0.068	3494	5.987	6.254	c
WT	60	3.24219	0.059	3494	3.126	3.358	a
GFP-SINE1	60	3.24389	0.067	3494	3.113	3.375	a
GFP-SINE1WIP1KASH #8	60	3.31572	0.064	3494	3.190	3.442	a
sine1-1	60	3.46389	0.059	3494	3.348	3.580	a
GFP-SINE1WIP1KASH #7	60	3.87557	0.065	3494	3.748	4.003	b
GFP-SINE1	120	3.11142	0.060	3494	2.994	3.229	a
WT	120	3.37126	0.059	3494	3.255	3.488	b
sine1-1	120	3.37681	0.059	3494	3.261	3.493	b
GFP-SINE1WIP1KASH #8	120	3.78886	0.052	3494	3.686	3.892	c
GFP-SINE1WIP1KASH #7	120	4.01208	0.057	3494	3.901	4.123	d
WT	180	2.58738	0.066	3494	2.458	2.716	a
GFP-SINE1	180	2.88492	0.051	3494	2.784	2.986	b
sine1-1	180	3.31603	0.057	3494	3.205	3.427	c
GFP-SINE1WIP1KASH #8	180	3.56223	0.060	3494	3.444	3.681	d
GFP-SINE1WIP1KASH #7	180	3.85298	0.060	3494	3.736	3.970	e

Journal of Cell Science • Supplementary information

Table S3E

Lines	p-value
(sine1-1) - WT	1
(GFP-SINE1) - WT	1
(GFP-SINE1) - (sine1-1)	4.0E-01
(GFP-SINE1WIP1KASH #7) - WT	5.1E-34
(GFP-SINE1WIP1KASH #7) - (sine1-1)	3.6E-40
(GFP-SINE1WIP1KASH #7) - (GFP-SINE1)	4.9E-28
(GFP-SINE1WIP1KASH #8) - WT	1.4E-18
(GFP-SINE1WIP1KASH #8) - (sine1-1)	1.4E-23
(GFP-SINE1WIP1KASH #8) - (GFP-SINE1)	2.1E-14
(GFP-SINE1WIP1KASH #8) - (GFP-SINE1WIP1KASH #7)	2.0E-03

Table S3F

Line	emmean	SE	df	LCL	UCL	group
WT	221.459	4.929	312	211.8	231.2	a
GFP-SINE1WIP1KASH #7	221.559	3.781	312	214.1	229.0	a
GFP-SINE1WIP1KASH #8	232.248	3.781	312	224.8	239.7	ab
sine1-1	238.529	3.674	312	231.3	245.8	b
GFP-SINE1	243.512	3.753	312	236.1	250.9	b

Table S3. Expanded statistics for Fig. 2 and Fig. S1. (A) p-values for stomatal opening (Fig. 2C and Fig. S1A). **(B)** Group assignments for stomatal opening (Fig. 2C and Fig. S1A). **(C)** p-values for stomatal closure (Fig. 2D and Fig. S1B). **(D)** Group assignments for stomatal closure (Fig. 2D and Fig. S1B). **(E)** p-values for stomatal area (Fig. S1D). **(F)** Group assignments for stomatal area (Fig. S1D). emmean – estimated marginal mean, LCL – lower confidence limit, UCL – upper confidence limit. P-values: red > 0.05, green ≤ 0.05.

Table S4. Expanded statistics for Fig. 3. (A) p-values for trichoblast circularity (Fig. 3C). **(B)** Group assignments for trichoblast circularity (Fig. 3C). **(C)** p-values for trichoblast area (Fig. 3D). **(D)** Group assignments for trichoblast area (Fig. 3D). emmean – estimated marginal mean, LCL – lower confidence limit, UCL – upper confidence limit. P-values: red > 0.05, green ≤ 0.05.

Table S4A

Lines	p-value
(GFP-WIP1) - WT	5.5E-05
(GFP-WIP1SINE1KASH4) - WT	4.8E-31
(GFP-WIP1SINE1KASH4) - (GFP-WIP1)	1.4E-15
(GFP-WIP1SINE1KASH5) - WT	1.2E-18
(GFP-WIP1SINE1KASH5) - (GFP-WIP1)	6.7E-06
(GFP-WIP1SINE1KASH5) - (GFP-WIP1SINE1KASH4)	1.3E-03
wip123 - WT	2.9E-24
wip123 - (GFP-WIP1)	5.9E-10
wip123 - (GFP-WIP1SINE1KASH4)	3.6E-01
wip123 - (GFP-WIP1SINE1KASH5)	7.5E-01

Table S4B

Line	emmean	SE	df	LCL	UCL	group
WT	0.43523	0.021	257	0.395	0.476	a
GFP-WIP1	0.57227	0.021	257	0.531	0.614	b
GFP-WIP1SINE1KASH5	0.72204	0.020	257	0.682	0.762	c
wip123	0.77408	0.021	257	0.733	0.815	cd
GFP-WIP1SINE1KASH4	0.83624	0.021	257	0.795	0.878	d

Journal of Cell Science • Supplementary information

Table S4C

Lines	p-value
wip123 - WT	4.2E-06
(GFP-WIP1) - WT	1
(GFP-WIP1) - wip123	8.9E-09
(GFP-WIP1SINE1KASH4) - WT	1.0E-05
(GFP-WIP1SINE1KASH4) - wip123	1
(GFP-WIP1SINE1KASH4) - (GFP-WIP1)	2.6E-08
(GFP-WIP1SINE1KASH5) - WT	4.2E-08
(GFP-WIP1SINE1KASH5) - wip123	1
(GFP-WIP1SINE1KASH5) - (GFP-WIP1)	4.7E-11
(GFP-WIP1SINE1KASH5) - (GFP-WIP1SINE1KASH4)	1

Table S4D

Line	emmean	SE	df	LCL	UCL	group
GFP-WIP1SINE1KASH5	54.1413	3.712	257	46.83	61.45	a
wip123	58.7207	3.747	257	51.34	66.10	a
GFP-WIP1SINE1KASH4	59.4363	3.819	257	51.92	66.96	a
WT	86.2434	3.747	257	78.87	93.62	b
GFP-WIP1	92.7814	3.819	257	85.26	100.3	b
